# A retrospective cohort study of the effectiveness and adverse events of intralesional pentavalent antimonials in the treatment of cutaneous leishmaniasis

**DOI:** 10.1016/j.ijpddr.2020.11.002

**Published:** 2020-11-19

**Authors:** Bruna Côrtes Rodrigues, Marina Freitas Ferreira, Daniel Holanda Barroso, Jorgeth Oliveira Carneiro da Motta, Carmen Déa Ribeiro de Paula, Cláudia Porto, Sofia Sales Martins, Ciro Martins Gomes, Raimunda Nonata Ribeiro Sampaio

**Affiliations:** a University Hospital of Brasília, Dermatology Department, SGAN 605, Asa Norte, Brasília-DF, 70840-901, Brazil; b Postgraduate in Medical Sciences, Medical College, University of Brasília, UnB - Darcy Ribeiro University Campus, Asa Norte, Brasília-DF, 70.910-900, Brazil; c Dermatomycology Laboratory, Medical College, University of Brasília, UnB - Darcy Ribeiro University Campus, Asa Norte, Brasília-DF, 70.910-900, Brazil; d Postgraduate in Health Sciences, Health Sciences College, University of Brasília, UnB - Darcy Ribeiro University Campus, Asa Norte, Brasília-DF, 70.910-900, Brazil

**Keywords:** Cutaneous leishmaniasis, New world leishmaniasis, Intralesional therapy, Intralesional antimonial, N-methyl glucamine, Pentavalent antimonial

## Abstract

**Introduction:**

The standard therapy for American cutaneous leishmaniasis (ACL) is intravenous meglumine antimoniate (IV-MA). However, treatment interruptions due to adverse events (AEs) and non-adherence are frequent. Consequently, intralesional MA (IL-MA) was proposed.

**Objective:**

This study examined the effectiveness of and AEs associated with IL-MA.

**Methods:**

We performed a retrospective cohort study of 240 patients with ACL. We excluded patients with mucous lesions and disseminated leishmaniasis and those who received treatment in the previous 6 months. We considered protocol treatments as the main risk factors. IL-MA was performed using a subcutaneous injection of MA in a volume sufficient to elevate the lesion base (approximately 1 mL/cm^2^ of lesion area) once weekly for 1–8 weeks. IV-MA was performed via intravenous injections of MA at a dosage of 10–20 mg Sb^5+^/kg/day for 20 days. The primary outcome was defined as a lesion cure 3 months after treatment, and AEs were secondary outcomes.

**Results:**

Seventy-three patients were included. The IL-MA group consisted of 21 patients, and the IV-MA group consisted of 52 patients. The IL-MA group was older, had more comorbidities and more previous unsuccessful treatment of ACL. The antimonial dose was significantly lower in this group. The cure rate for IL-MA was 66.7%, which was lower than that in the IV-MA group (relative risk (RR) = 0.68, 95% CI: 0.50–0.92, p < 0.001), while the rate of AEs was similar. Female sex (RR = 1.16, 95% CI: 1.02–1.33), lesion diameter ≤1 cm (RR = 1.25, 95% CI: 1.00–1.56) and treatment with IV-MA (RR = 1.43, 95% CI: 1.06–1.93) were independently associated with achieving a cure. Comorbidities (RR = 1.7, 95% CI: 1.06–2.98) were independently associated with AEs.

**Conclusions:**

Patients of IL-MA group were older, had more comorbidities and more previous unsuccessful treatment of ACL. Nevertheless, IL-MA had a cure rate of 66.7%, and it was useful in this context. A prospective randomized trial is recommended.

## Highlights

• Intralesional antimonials (IL-MA) had a 66.7% lower cure compared to systemic.
• IL-MA group was older, had more comorbidities and unsuccessful previous treatment.
• IL-MA was useful in this group, where worse clinical responses are expected.
• Lesions larger than 3 cm did not have more chance of failure with IL-MA.
• There was no mucous recurrence after IL-MA in an area of New World Leishmaniasis.

## Graphical abstract

**Figure:**
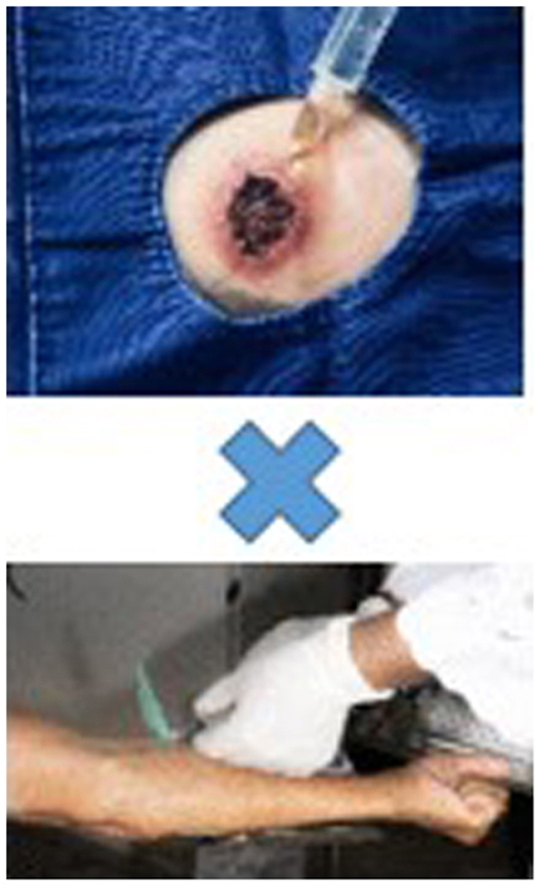

## 1. introduction

1

American cutaneous leishmaniasis (ACL) is a neglected tropical disease caused by a protozoa of the genus *Leishmania*, which are transmitted through the bite of a phlebotomine sandfly (Bates and Rogers, 2004). The most important species causing ACL in Brazil are *Leishmania (Viannia) braziliensis*, *Leishmania (Leishmania) amazonensis* and *Leishmania (Viannia) guyanensis* (Brasil, 2017). Clinical manifestations depend on the interaction between the parasite and the host's immune response (Mitropoulos et al., 2010). ACL generally affects the skin, but it may progress to late mucosal involvement, and it has a low spontaneous cure rate (Cota et al., 2016). Treatment is necessary because it represents the first measure of disease control.

The standard therapy for ACL is a pentavalent antimonial, such as meglumine antimoniate (MA), administered parenterally at a dose of 10–20 mg Sb^5+^/kg/day for 20 days (Lima et al., 2007; Brasil, 2017; PAHO/WHO, 2019). This therapy is associated with several adverse events (AEs). The most common AEs are myalgia, arthralgia, fatigue, anorexia, nausea and headache (Brasil, 2017). Severe and potentially fatal AEs may occur, such as pancreatitis, hepatitis, arrhythmias and renal toxicity (Oliveira et al., 2011; Lyra et al., 2016). Pregnant women, elderly individuals and patients with comorbidities or coinfection may require a different approach (Brasil, 2017).

Treatment modalities to reduce toxicity, improve efficacy and facilitate administration and adherence have emerged for the treatment of ACL in recent decades. Injections of intralesional MA (IL-MA) have been performed in reference centres in Brazil for more than 20 years. The Pan American Health Organization (PAHO) included IL-MA as an alternative therapy via consensus in 2013 and emphasized the low level of evidence (PAHO/WHO, 2019). The Ministry of Health of Brazil recommends IL-MA for patients with ACL, including recidiva cutis, with a single lesion up to 3 cm in its largest diameter, in any location, except for the head and periarticular regions, and without immunosuppression (Brasil, 2017). There are no controlled trials that indicate the efficacy of this therapy in the treatment of ACL (Brito et al., 2017), which means that observational data are important for future research.

The present study evaluated the ACL cure and AEs rates in patients who received IL-MA compared to patients who received intravenous MA (IV-MA).

## 2 Materials and methods

### 2.1 Population and case definition

We performed a retrospective cohort study and included ACL patients treated with MA at the University Hospital of Brasília, Brazil, from 1999 to 2017. ACL case definition relied on clinical, laboratory and epidemiological criteria described elsewhere (Gomes et al., 2014). ACL was also confirmed via parasitological confirmation as successful culture, polymerase chain reaction or histopathology. We excluded patients with mucous lesions, patients with disseminated or diffuse cutaneous leishmaniasis, patients who received treatment 6 months prior to the main evaluation and patients who were lost to follow-up within 3 months after treatment.

### 2.2 Sampling

A sample calculation was performed using SAS 9.4 software considering a difference in the percentage of cure between the two groups of 34% (Vasconcellos, 2013; Carvalho et al., 2019). For the IV-MA group, we used a cure rate of 90% based directly on the cure proportions described by Carvalho et al. (2019), which was from 60 to 90% (Carvalho et al., 2019). We used the highest cure rate shown by the authors once, in the present hospital, we used the highest antimonial dosage defined as 20 mg Sb^5+^/kg/day or a daily maximum of 15 mL. For the IL-MA group, we used a cure rate of 56%. This rate was justified by the scarcity of existing data and a level of arbitration based on our clinical expertise. Vasconcellos (2013) described that 18 of 32 relapsed patients were cured using IL-MA (Vasconcelos, 2013). The set of 32 patients in the present study received a variety of treatment combinations using systemic MA. Other patients started parenteral treatment before IL-MA, but we considered the total sample of 32 patients for the sample size calculation because it is also described that IL-MA may have systemic effects. Therefore, the effect of IL-MA treatment at its lower confidence interval will require more powerful outcomes to achieve statistical significance. It was estimated that with a sample of 73 patients, 24 of whom were treated with IL-MA and 49 treated with IV-MA, the study would have 90% power to detect differences clinically between groups about the percentage of cure, for a significance level of 5%.

**Fig. 1:**
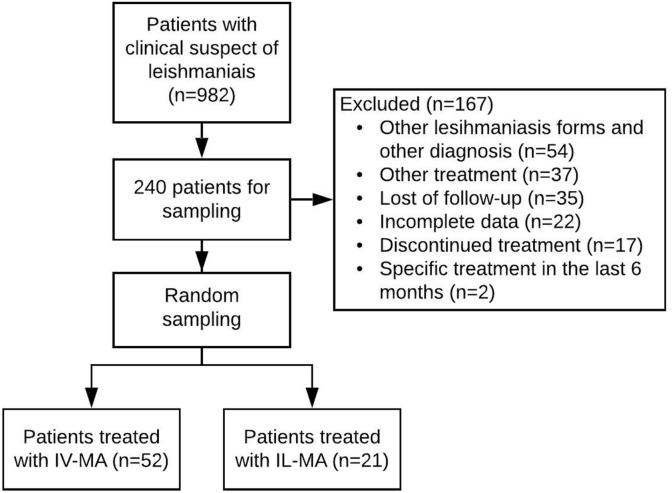
Flowchart of patient selection. Legend: IV-MA = Intravenous meglumine antimoniate; IL-MA: Intralesional meglumine antimoniate.

Our main register located 982 patients who could potentially fulfil the pre-defined inclusion criteria. The HUB recently started the use of an electronic register for consultation. Older patient files are still stored in paper files, and those files are kept in warehouse located 15 km from the hospital, which makes accessing them a challenging bureaucratic task. Due to complete files are difficult to access, we used a list of identification data (simple registers, ambulatory and laboratorial data) that allowed a first screening of 240 eligible patients, which was part of our target population. Due to these internal difficulties and to make the study feasible, we used a well-validated random sampling technique to provide a picture of the population profile (cure rate) with a 90% power at a significance level of p < 0.05 (Fig. 1).

### 2.3 Risk factors

The main risk factor analysed in this study was the use of IL-MA. There is no consensus on the best technique for this treatment or restrictions relating to the number, size and location of the lesions. IL-MA was performed in ACL patients who had up to 2 lesions in this centre. The institutional intralesional protocol was based on Gadelha et al. (1990) and Aste et al. (1998) (Gadelha et al., 1990; Aste et al., 1998). It involved a subcutaneous injection of MA in a sufficient volume to elevate the lesion base (approximately 1 mL/cm^2^ of lesion area), with a maximum of 5 mL, once weekly for 1–8 weeks. Alternatively, IV-MA therapy was performed using intravenous 10–20 mg Sb^5+^/kg/day for 20 days according to the PAHO and Ministry of Health of Brazil recommendations (Brasil, 2017; PAHO/WHO, 2019). No specific local care was established in this centre.

Other variables were also analysed, such as demographic characteristics, comorbidities, number, size and localization of lesions, antimonial cumulative dose (mg Sb^5+^/kg/day), treatment duration, and interruption time. Treatment interruption was considered when patients stopped treatment for over one week in the IL-MA group based on institutional protocol, and over one day in the IV-MA group or once the antimony half-life was approximately 32.8 ± 3.8 h (Gomes et al., 2015).

### 2.4 Outcomes

The primary outcome was defined as leishmaniasis cure (epithelized lesion 3 months after treatment). If this criterion was not fulfilled, the patient was considered not cured at this time, and a new treatment was performed, according to a routine protocol. The occurrence of AEs was considered a secondary outcome. We classified AEs according to their potential effect on treatment continuation: mild: patient should be closely monitored (most commonly myalgia, arthralgia, local inflammation, headache); and severe: treatment must be interrupted immediately (most commonly pancreatic involvement, cardiotoxicity, nephrotoxicity and hepatotoxicity). According to institutional routine, patients were monitored weekly during treatment with MA to identify AEs, which were characterized as clinical, laboratory and electrocardiographic changes that occurred during treatment, with no possible causal relationship to external factors.

### 2.5 Statistical analysis

Cure and AEs were considered individually as dependent variables. The independent variables were sex (male; female), age (≤50; >50 years), duration of lesion (≤4; >4 months), number of lesions (≤1; >1), size of lesion (≤1; >1 cm), antimonial dose (≤10; >10 mg Sb^5+^/kg/day), group (IL-MA; IV-MA), comorbidities, interruption, cure and AEs, with no or yes answers. The determination of the age cut-off was based on recommendations from the Ministry of Health (Brasil, 2017). The other numerical cut-offs were determined by the median of the values of each respective variable.

The Poisson regression model with robust variance was used to test the effect of independent variables on the occurrence of AEs and cure. The statistical analysis consisted of obtaining the frequencies, incidences and confidence intervals of the independent variables. A bivariate analysis was performed, and the association between each independent variable and the occurrence of cure or AEs was verified. Results with p < 0.25 were selected for multivariate analysis.

The multiple analysis models were constructed by consecutively excluding a variable with the highest p-value from the Wald test (Hosmer et al., 2013), with subsequent readjustment and stability verification. The variables that were excluded were added to the final model, one by one, and the Poisson regression analysis was repeated. Only variables with p < 0.05 remained in the final model. Multicollinearity of the independent variables was evaluated. The limit of the presence of multicollinearity was considered when the tolerance indicator assumed values lower than 0.4. Statistical significance was defined as a p value of 0.05, and CIs were set at 95%. Statistical analyses were performed using SAS 9.4 Software.

### 2.6 Methodological limitations

It is important to highlight that retrospective studies have limitations, such as missing data in medical records, difficulty in standardizing the technique and characteristics of the population and loss to follow-up.

## 3 Results

Seventy-three randomly selected patients were included in this study. The IL-MA group consisted of 21 patients, and the IV-MA group consisted of 52 patients.

### 3.1 Demographic and clinical characteristics

Table 1 shows the demographic characteristics and clinical features of the patients in both groups. There were similarities in the duration of disease, size of lesion, weight, gender predominance (male) and incidence of lower limb lesions. However, the IL-MA patients were older, had a lower number of lesions and had a higher prevalence of comorbidities compared to the IV-MA patients. There were no facial lesions in the patients in the IL-MA group. The IL-MA patients also had a higher prevalence of a poor response to previous systemic treatment. The lesion diameter ranged from 1 to 7 cm in both groups.

**Table 1:** Demographic characteristics and clinical features of the patients in the 2 treatment groups at baseline.

Variables	IL-MA group (n = 21)	IV-MA group (n = 52)	P value
	Mean (SD)	Mean (SD)	
**Age of patients (years)**	49.86 (21.34)	33.12 (18.81)	0.003
**Duration of lesions (months)**	5.01 (5.21)	6.90 (14.52)	0.798
**Number of lesions**	1.10 (0.30)	1.90 (1.56)	0.006
**Size of lesions (cm)**	3.11 (1.84)	3.70 (2.45)	0.316
**Weight (kg)**	60.00 (5.37)	60.89 (18.62)	0.608
	**N (%)**	**N (%)**	
**Sex**			
Male	16 (76.20)	36 (69.20)	0.552
Female	5 (23.80)	16 (30.80)	
**Single lesion**	19 (90.50)	29 (55.77)	0.006
**Lower limb lesion**	11 (52.40)	22 (42.30)	0.450
**No previous treatment for ACL**	18 (85.70)	52 (100.00)	0.021
**Comorbidities**	11 (52.40)	11 (21.20)	0.008

Legend: IL-MA = intralesional meglumine antimoniate, IV-MA = intravenous meglumine antimoniate, ACL = American cutaneous leishmaniasis, SD = standard deviation, n = number of patients.

### 3.2 Treatment characteristics

3.2

Table 2 shows comparisons of treatment data between the 2 groups. Treatment duration was longer in the IL-MA group. The number of weekly sessions in this group ranged from 2 to 8 (mean 4.33). The dose of MA was lower in the IL-MA group. The volume of MA ranged from 1 to 5 mL per session (mean 2.88 mL) in the IL-MA group, and it ranged from 4 to 15 mL per day (mean 12.7 mL) in the IV-MA group. There was no significant difference in treatment interruption between groups. The interruptions were all transient in the IV-MA group, but 3 patients (14.29%) in the IL-MA group had definite interruptions due to cardiotoxicity, although one of them was healed.

**Table 2:** Comparison of treatment data between the 2 groups.

Variables	IL-MA group (n = 21)	IV-MA group (n = 52)	P value
	Mean (SD)	Mean (SD)	
**Treatment duration (days)**	30.33 (9.73)	20.00 (0.00)	<0.001
**Interruption duration (days)**	8.11 (14.05)	2.06 (4.23)	0.153
**Treatment and interruption duration (days)**	37.67 (22.08)	22.06 (4.23)	<0.001
**Antimonial total dose (mg)**	1012.50 (613.95)	20574.78 (5237.45)	<0.001
**Antimonial dose (mg/kg/day)**	0.48 (0.23)	17.13 (2.96)	<0.001
	**N (%)**	**N (%)**	
**Treatment interruption**	9 (42.90)	13 (25.00)	0.132
**Cure**	14 (66.70)	51 (98.10)	<0.001
**Adverse events**	11 (52.40)	28 (53.80)	0.91
Mild^a^	7 (33.33)	17 (32.69)	1.00
Severe^b^	4 (19.05)	11 (21.15)	1.00

Legend: IL-MA = intralesional meglumine antimoniate, IV-MA = intravenous meglumine antimoniate, SD = standard deviation, n = number of patients.

a Mild = monitor closely.

b Severe = interrupt treatment.

The cure rate was significantly lower in the IL-MA group than the IV-MA group. Patients with upper limb lesions in the IL-MA group were more frequently cured than patients who had lesions elsewhere (p = 0.018). The number and size of lesions, treatment duration, antimonial dose and other variables in the IL-MA group were not associated with a higher chance of cure or AEs. The risk of AEs was not different between groups. The classification of AEs as mild and severe was also not different (Table 2). Among the 11 IL-MA patients who experienced AEs (52.38%), 7 (33.33%) suffered local inflammation, 3 (14.29%) developed cardiotoxicity (enlargement of corrected QT interval (QTc) on an electrocardiogram (EKG), bradycardia and T-wave inversion and sinus tachycardia and extrasystoles), 2 (9.52%) suffered headache, 2 (9.52%) developed myalgia and 1 (4.76%) exhibited elevation of hepatocellular enzymes. Among the 28 IV-MA patients who experienced AEs (53.80%), 11 (21.15%) developed myalgia, 7 (13.46%) exhibited arthralgia, 4 (7.69%) suffered local inflammation, 4 (7.69%) exhibited amylase elevation, 4 (7.69%) had headache, 4 (7.69%) exhibited cardiotoxicity, 2 (3.85%) suffered nephrotoxicity and 2 (3.85%) had transaminase elevation. Seven patients (33.33%) treated with IL-MA had lesions over 3 cm, in disagreement with current Ministry of Health guidelines. However, the cure rate and AEs rate were not different compared to the other patients in this group.

### 3.3 Cure – multivariate analysis

There was a significant association between the occurrence of cure and the following variables: female sex, lesion diameter ≤1 cm, antimonial dose ≥10 mg Sb^5+^/kg/day and the application of IV-MA (Table 3). The tolerance indicator for multicollinearity ranged from 0.60 to 0.95, which shows that there was no strong multicollinearity between the independent variables. The multivariate analysis showed that female patients had a 16% higher chance of cure. Lesions with diameters ≤1 cm had a 25% greater chance of cure, and patients treated with IV-MA had a 43% greater chance of cure (Table 3).

**Table 3:** Cure rate according to variables and distribution of study variables according to gross and adjusted relative risk as calculated using a Poisson regression model with robust variance and their respective 95% confidence intervals. Thirteen subjects with missing values were excluded.

				Gross RR		Adjusted RR	
Variables	Frequency (n = 60)	Cure Rate (%)	95%CI	RR (95%CI)	P value	RR (95%CI)	P value
**Sex**					0.008		0.023
Male	44	84.09	72.96–95.22	1	–	1	–
Female	16	100.00	100.00–100.00	1.19 (1.05–1.35)	0.008	1.16 (1.02–1.33)	0.023
**Age (years)**					0.431		–
≤50	43	90.70	81.76–99.64	1.10 (0.87–1.40	0.431	–	–
>50	17	82.35	63.70–100.00	1	–	–	–
**Duration of lesions (months)**					0.254		–
≤4	42	85.71	74.82–96.61	1	–	–	–
>4	18	94.44	83.55–100.00	1.10 (0.93–1.30)	0.254	–	–
**Number of lesions**					0.620	–	–
≤1	38	86.84	75.78–97.91	1	–	–	–
>1	22	90.91	78.54–100.00	1.05 (0.87–1.25)	0.620	–	–
**Size of lesions (cm)**					0.008		0.048
≤1	6	100.00	100.00–100.00	1.15 (1.04–1.27)	0.008	1.25 (1.00–1.56)	0.048
>1	54	87.04	77.81–96.26	1	–	1	–
**Antimonial dose (mg/kg/day)**					0.025	–	–
≤10	19	68.42	46.90–89.94	1	–	–	–
>10	41	97.56	92.70–100.00	1.43 (1.05–1.94)	0.025	–	–
**Treatment interruption**					0.385	–	–
No	44	90.91	82.16–99.65	1.12 (0.87–1.44)	0.385	–	–
Yes	16	81.25	61.56–100.00	1	–	–	–
**Adverse events**					0.688		–
No	30	86.67	30	1	–	–	–
Yes	30	90.00	30	1.04 (0.86–1.25)	0.688	–	–
**Comorbidities**					0.988	–	–
No	43	88.37	43	1.00 (0.82–1.23)	0.988	–	–
Yes	17	88.23	17	1	–	–	–
**Group**					0.025		0.018
IL-MA	19	68.42	19	1	–	1	–
IV-MA	41	97.56	41	1.43 (1.05–1.94)	0.025	1.43 (1.06–1.93)	0.018

Legend: IL-MA = intralesional meglumine antimoniate, IV-MA = intravenous meglumine antimoniate, RR = relative risk, CI = confidence interval, n = number of patients.

Note: There was no significant association between cure and the variables age, duration of lesions, number of lesions, antimonial dose, treatment interruption, adverse events and comorbidities in the bivariate and multivariate analyses.

### 3.4 Adverse events – multivariate analysis

There was a significant association between the occurrence of AEs and the following variables: treatment interruption and the presence of comorbidities (Table 4). The tolerance indicator for multicollinearity ranged from 0.60 to 0.95, which indicates that there was no strong multicollinearity between the independent variables. Patients with comorbidities had 78% more risk of exhibiting AEs than healthy patients (Table 4).

**Table 4:** Adverse event rate according to variables and the distribution of study variables according to gross and adjusted relative risk as calculated using a Poisson regression model with robust variance and their respective 95% confidence intervals. Thirteen subjects with missing values were excluded.

				Gross RR		Adjusted RR	
Variables	Frequency (n = 60)	Cure Rate (%)	95%CI	RR (95%CI)	P value	RR (95%CI)	P value
**Sex**					0.211		–
Male	44	45.45	30.31–60.60	1	–	–	–
Female	16	62.50	38.08–86.92	1.37 (0.83–2.26)	0.211	–	–
**Age (years)**					0.124		–
≤50	43	44.19	28.90–59.47	0.68 (0.42–1.11)	0.124	–	–
>50	17	64.71	41.32–88.09	1	–	–	–
**Duration of lesions (months)**					1.000		–
≤4	42	50.00	34.43–65.57	1	–	–	–
>4	18	50.00	26.22–73.78	1.00 (0.58–1.74)	1.000	–	–
**Number of lesions**					1.000	–	–
≤1	38	50.00	33.63–66.37	1	–	–	–
>1	22	50.00	28.49–71.51	1.00 (0.59–1.69)	1.000	–	–
**Size of lesions (cm)**					0.204	–	–
≤1	6	16.67	0.00–47.37	3.22 (0.53–19.62)	0.204	–	–
>1	54	53.70	40.01–67.40	1	–	–	–
**Antimonial dose (mg/kg/day)**					0.785	–	–
≤10	19	47.37	24.25–70.48	1	–	–	–
>10	41	51.22	35.47–66.97	1.08 (0.62–1.89)	0.785	–	–
**Treatment interruption**					0.001	–	0.049
No	44	38.64	23.82–53.45	1	–	1	–
Yes	16	81.25	61.56–100.00	2.10 (1.35–3.27)	0.001	1.60 (1.00–2.63)	0.049
**Cure**					0.705		–
No	7	42.86	5.11–80.60	1	–	–	–
Yes	53	50.94	37.09–64.80	1.19 (0.49–2.91)	0.705	–	–
**Comorbidities**					0.001		0.029
No	43	37.21	22.33–52.08	1	–	1	–
Yes	17	82.35	63.70–1000	2.21 (1.42–3.46)	0.001	1.78 (1.06–2.98)	0.029
**Group**					0.785		–
IL-MA	19	47.37	24.25–70.48	1	–	–	–
IV-MA	41	51.22	35.47–66.97	1.08 (0.62–1.89)	0.785	–	–

Legend: IL-MA = intralesional meglumine antimoniate, IV-MA = intravenous meglumine antimoniate, RR = relative risk, CI = confidence interval, n = number of patients.

Note: There was no significant association between adverse events and the variables sex, age, duration of lesions, number of lesions, size of lesions, antimonial dose, cure rate and treatment group in the bivariate and multivariate analyses.

## 4 Discussion

These results were obtained from years of experience with intralesional therapy for ACL in a Brazilian reference centre, but there were some methodological limitations. The data were derived from only one centre, with a small number of subjects and limited follow-up. It was not possible to compare equal MA doses and treatment duration between groups. It was also not possible to standardize the IL-MA technique and the characteristics of patients, such as age and the presence of comorbidities.

As previously discussed, the main treatment for ACL is the systemic use of Sb^5+^, which involves daily visits to the health unit for at least 20 days. For many populations, daily access may be expensive and often results in treatment abandonment. The intralesional use of Sb^5+^ in this context has greater flexibility because the patient appears less frequently in the health unit. The occurrence of serious AEs with systemic Sb^5+^ may lead to an absence from work and consequent economic impact. The use of a lower dose of Sb^5+^ in IL-MA reduces potential serious AEs, which makes this treatment an option for more vulnerable populations, such as patients with comorbidities (Duque et al., 2019; Rodriguez et al., 2019). This context led us to compare intravenous MA to the intralesional use of MA, which use the same drug but differ in treatment dose and duration.

IL-MA emerged in the Americas initially as an alternative therapy for patients with ACL with clinical or social conditions that complicate the use of systemic antimonials (Oliveira-Neto et al., 1997). The indication for intralesional therapy in 58% of the patients in other referral centres was the presence of one or more contraindications to systemic antimony treatment, including advanced age and comorbidities (Vasconcellos et al., 2012; Silva et al., 2016). The IL-MA group exhibited increased median age, comorbidity prevalence and rate of previous systemic ACL compared to the IV-MA group. These differences reflect the main historical indication of this therapy as an alternative treatment for specific groups. The higher rate of previous ACL treatment may indicate intolerance with previous systemic therapy, which makes it difficult to complete the treatment. Therefore, IL-MA may have been indicated because of its potentially improved safety profile. Many of the patients who completed treatment with IL-MA would have contraindications for IV-MA or would not have completed this treatment. However, worse results were expected for AEs and the therapeutic response in the IL-MA group because of these differences in clinical and demographic characteristics. These patients were not homogenous, and comparisons of results must be done with caution. Therefore, a cure rate of 67% and the similarity between AEs was a good result for us in the context of the characteristics of the IL-MA group participants.

The decreased number of lesions in the IL-MA group also reveals a historical tendency of the indications for the use of this therapy in localized disease, as described elsewhere (Silva et al., 2016). The local institutional protocol includes IL-MA as a treatment possibility for patients with up to 2 lesions. However, there is no consensus, and there are reports of successful treatment in patients with a higher number of lesions (Duque et al., 2019). The PAHO (2013) included intralesional antimonial application as a possible treatment for localized ACL, which was characterized as one lesion up to 3 cm diameter, except lesions localized on the face or joints (PAHO/WHO, 2013). One interesting result of our study was that 7 patients treated with IL-MA had lesions larger than 3 cm, and no differences in cure rates or AEs was found compared to the subgroup with lesions smaller than 3 cm. Other authors reported a good response and safety with this local treatment in patients with lesions larger than 3 cm (Oliveira-Neto et al., 1997; Vasconcellos et al., 2012; Pimentel et al., 2017; Duque et al., 2019). This fact may indicate that the size restriction in the ACL guidelines (Brasil, 2017; PAHO/WHO, 2019) may be revised in the future due to a lack of scientific evidence to support this restriction.

Some studies showed that lesions located on the lower limbs needed more time to heal, and venous stasis may be a delaying factor (Schubach et al., 2005; Silva et al., 2018). Although the rate of lower limb lesions was not different between groups, patients in the IL-MA group with lesions on the lower limbs had a lower rate of cure than patients with upper limbs lesions. The IL-MA group was older and had more comorbidities, and likely had more circulatory disturbances in lower limbs, which supports the difficulty in lesion healing in that location.

Women generally present with more resistant and severe lesions and require higher doses of antimonials than men because oestrogen is related to the increased production of IL4 and IL10, which inhibit IFN-alpha production and macrophage activation (Baccan et al., 2011; Conceição-Silva et al., 2018). However, women exhibited a higher rate of cure than men in the present study.

One advantage of intralesional therapy is the low antimonial dose, which is administered on a convenient schedule without the necessity of daily injection (Aguiar et al., 2018). The IL-MA group in the present had a longer treatment duration than the IV-MA group. However, the number of visits to the health unit in the IL-MA group was lower. The protocol with systemic antimonials involves daily visits to the health unit for 20 days, but the protocol for IL-MA allows a reduction in the number of visits with a larger interval, which favours the adherence to treatment, especially for patients with unfavourable socio-economic conditions, as cited elsewhere (Duque et al., 2019; Rodriguez et al., 2019). The IL-MA group received a lower total dose of antimonials than the IV-MA group, as expected. This variable was not associated with the rates of cure or AEs in the multivariate analysis or the individual group analysis. The differences in baseline characteristics seemed to have a more important influence on these outcomes.

The intralesional protocol followed in the present study was a weekly injection of MA for 1–8 weeks. The first report of this technique was used for Old World leishmaniasis, and the most common published injection interval was 7 days (range 3–7 days) for up to 10 sessions (Brito et al., 2017). In New World leishmaniasis studies, injection intervals ranged from 1 to 15 days for up to 10 sessions (Brito et al., 2017). The PAHO recommendations are 1–5 infiltrations of 1–5 mL of MA per session, every 3–7 days (PAHO/WHO, 2019). The Ministry of Health of Brazil recommends 1 to 3 infiltrations of approximately 5 mL of MA per session, with an interval of 15 days (Brasil, 2017). This heterogeneity between studies reflects the lack of consensus on the best technique for IL-MA treatment in the number of doses, time interval between doses and volume of injected IL-MA. This lack of definition hinders comparisons with previous data. Therefore, the present did not observe an influence of antimonial dose or duration of IL-MA treatment on the outcomes cure and AEs.

There was no difference in the prevalence of interruption between the groups in the present study. Most reasons for treatment discontinuation were due to AEs and rarely due to adhesion problems. The institutional protocol in this reference centre was weekly follow-up during treatment. This schedule had a positive influence on patient adherence to both treatments. We did not observe a difference in AEs prevalence or level between the groups. We observed changes in cardiac and hepatic tests during treatment with IL-MA. Antimonial intralesional absorption occurs (Neves et al., 2009), and it is as efficient as intramuscular administration (Aguiar et al., 2018). Laboratory and electrocardiographic changes, including hepatic, renal and haematological impairment and enlargement of QTc on EKG, were reported in IL-MA patients (Neves et al., 2009; Vasconcellos et al., 2012). Although the AEs associated with antimonial treatment are generally dose-dependent (Marsden, 1985; Neves et al., 2009), we did not observe an association between total dose and the occurrence of AEs. Notably, the IL-MA and IV-MA groups were not fully comparable because patients in these groups had different clinical and demographic characteristics, such as age, comorbidities and the prevalence of comorbidities, which likely influenced the AEs results. Multivariate analysis revealed that the presence of comorbidities was an independent variable associated with AEs, which confirmed our hypothesis.

The cure rate of IV-MA in our study was 98.1%. A previous ACL meta-analysis reported a cure rate of 76.9% (Tuon et al., 2008), and the rate is generally in the range of 60–90% (Carvalho et al., 2019). However, a cure rate of 94.4% for ACL treated with IV-MA was described (Saheki et al., 2017). Despite this variability in the literature data, our cure rate with IV-MA was higher than expected. The IL-MA group had a cure rate of 66.70%. A recent systematic review of the intralesional technique showed an efficacy of 77% in ACL patients, but different treatment regimens (interval, length of treatment and number of doses injected) make a direct comparison difficult (Brito et al., 2017). Previously, IL-MA treatment longer than 14 days was associated with cure (Brito et al., 2017). There was no association between cure and treatment duration in the IL-MA group in our study. Notably, the IL-MA group had some characteristics that may have negatively influenced the cure rate in our study, such as higher age, prevalence of comorbidities and the previous failure of IV-MA treatment. Therefore, a cure rate of 66.7% in this population was seen as a good result. These patients would likely not have received additional antimonial treatment without the use of the intralesional technique. Another important topic is the cure criteria. The present study evaluated cure after 90 days, but some studies evaluated the definitive cure after 360 days (Saheki et al., 2017; Duque et al., 2019). This difference may influence our results because some patients could exhibit a late healing and would have been considered cured if the cure criteria was extended. A definitive cure criteria of 180 days after treatment was proposed previously to standardize ACL studies (Olliaro et al., 2018).

The subjects of this retrospective study were followed-up for at least 3 months, but 36 (69.23%) patients in the IV-MA group and 13 (61.9%) patients in the IL-MA group had a follow-up of at least 1 year. Twenty-six (50%) of the patients in the IV-MA group and 10 (47.62%) patients in the IL-MA group had a follow-up of at least 2 years. Ten (19.23%) of the patients in the IV-MA group and 2 (9.52%) patients in the IL-MA group were followed for more than 5 years. No mucosal or recurrent cutaneous leishmaniasis was reported in the patients of this study. Other studies of ACL also showed no mucosal involvement after intralesional therapy in long-term follow-up (Oliveira-Neto et al., 1997; Vasconcellos et al., 2012; Brahim et al., 2017; Duque et al., 2019; Limachi-Choque et al., 2020). The limitations of a retrospective study, including missing data and loss to follow-up, may have influenced the results.

The intralesional technique had a lower cure rate than the intravenous antimonial technique and a similar AEs rate in this cohort study. However, the IV-MA and IL-MA groups had different baseline characteristics: patients treated with IL-MA were older, had more comorbidities and more previous ACL treatment attempts. Therefore, it was expected that this group would have a lower cure rate and increased risk of AEs. The IL-MA group had a cure rate of 66.7% and similar AEs as the IV-MA group, which is a good result for this special population. Nevertheless, IL-MA is a useful treatment option for elderly patients, patients with comorbidities and patients with other unsuccessful specific systemic treatment attempts. No progress to mucosal injury was observed in the patients follow-up. A prospective randomized trial with a representative sample is recommended to further evaluate this practical technique.

## Declaration of competing interest

None.
